# Metabolic crosstalk among cancer-associated fibroblasts, adipocytes and immune cells as an immunosuppressive tumor microenvironment driver

**DOI:** 10.1038/s12276-026-01650-1

**Published:** 2026-02-13

**Authors:** Tae Hyun Kim, Seong Hun Lim, Hyesung Lee, Young Chan Chae, Do Sik Min

**Affiliations:** 1 Department of pharmacy, Yonsei University, Incheon, Republic of Korea; 2 Department of Biological Sciences, Ulsan National Institute of Science and Technology, Ulsan, Republic of Korea

**Keywords:** Cancer metabolism, Cancer microenvironment

## Abstract

The tumor microenvironment (TME) is a complex ecosystem composed of not only malignant cells but also diverse stromal and immune cell populations that collectively shape tumor behavior. Metabolism is a central regulator of the TME, orchestrating intercellular communication through altered nutrients and signaling pathways to influence both the metabolic plasticity of cancer cells and functional balance of immune populations, ultimately determining tumor progression and antitumor immunity. Although tumor-intrinsic metabolic programs have been extensively characterized, emerging evidence highlights stromal metabolism as the dominant force sculpting immune responses within the TME. Among the nonmalignant stromal constituents, cancer-associated fibroblasts and cancer-associated adipocytes have emerged as metabolically active hubs that release and redistribute key metabolites, such as lactate, fatty acids and amino acids, to modulate the activity of both tumor and immune cells. Here we integrate recent advances in the understanding of stromal–immune metabolic crosstalk and elucidates how diverse metabolic mechanisms, including nutrient competition, mitochondrial remodeling, redox imbalance and immunometabolic rewiring, collectively reinforce an immunosuppressive TME and drive therapeutic resistance. Our study highlights the emerging strategies for selectively reprogramming these metabolic networks as potential therapeutic avenues. Deciphering these multilayered interactions will establish a conceptual and mechanistic foundation for reprogramming TME, restoring immune competence and enhancing the efficacy of current immunotherapies through metabolism-targeted interventions.

## stromal metabolism shapes tumor immunity and progression

Cancer is not just a disease of cancerous cells but involves a complex environment around the tumor. This environment includes various cells and structures that interact with cancer cells, affecting how the cancer grows and responds to treatment. The study addresses how this environment, particularly cancer-associated fibroblasts (CAFs) and adipocytes (CAAs), influences cancer progression. Researchers found that CAFs and CAAs are not just passive structures but actively change their metabolism to support cancer growth. They conducted a review of existing studies to understand these interactions better. CAFs often increase glycolysis and produce lactate, which helps cancer cells grow. CAAs release fatty acids that fuel cancer cells and suppress immune responses. The study concludes that targeting these metabolic changes in CAFs and CAAs could improve cancer treatments by restoring immune function and reducing tumor growth.

This summary was initially drafted using artificial intelligence, then revised and fact-checked by the author.

## Introduction

**Fig. 1:**
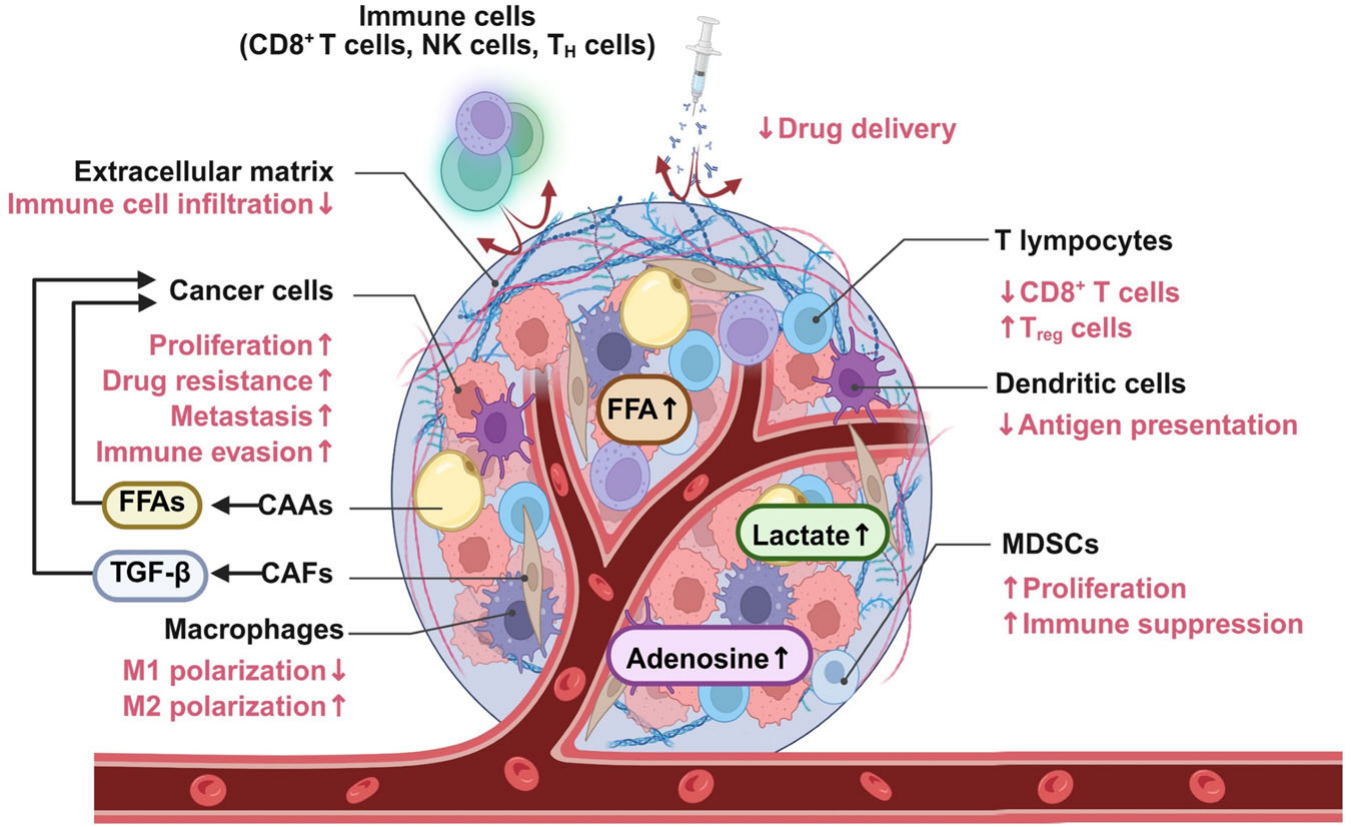
Metabolic crosstalk and immunosuppressive signaling in TME. The TME comprises a diverse network of cellular constituents including malignant cells, CAFs, CAAs, macrophages, MDSCs, dendritic cells and T lymphocytes. Both cancer cells and stromal elements such as CAFs and CAAs undergo profound metabolic reprogramming, leading to the release of immunomodulatory metabolites and signaling mediators, including FFAs. Lactate and adenosine levels: these metabolites not only serve as bioenergetic and biosynthetic substrates but also act as potent signaling cues that drive tumor progression. Mechanistically, they promote cancer cell proliferation, survival, metastatic dissemination and therapeutic resistance, while concurrently impairing antitumor immunity through the suppression of cytotoxic T and NK cell function, expansion of T_reg_ and polarization of TAMs toward an immunosuppressive phenotype. This coordinated metabolic-immune axis positions stromal–tumor metabolic crosstalk as a central driver of immune evasion and is a critical therapeutic target.

Cancer is not merely a disorder of autonomous malignant cells but a complex, systems-level pathology that evolves within the tumor microenvironment (TME), which is a dynamic and heterogeneous ecosystem composed of both cellular and structural components. Along with malignant cells, the TME encompasses cancer-associated fibroblasts (CAFs), cancer-associated adipocytes (CAAs), diverse immune cell subsets, vascular networks and extracellular matrix (ECM). These elements do not passively coexist; rather, they engage in continuous bidirectional communication that orchestrates fundamental processes such as tumor initiation, progression, metastasis, immune evasion and therapeutic resistance^1,2^. Central to these interactions is metabolic reprogramming, a well-established hallmark of cancer and a key driver of tumor adaptation, survival and malignant progression. Both tumor cells and their surrounding stromal and immune counterparts, including CAFs, CAAs and infiltrating immune cells, dynamically reconfigure their metabolic pathways to meet the evolving demands of the TME^3,4^. Although cancer metabolism has traditionally been examined in the context of fulfilling energetic and biosynthetic requirements, metabolites such as lactate, adenosine and free fatty acids (FFAs) also act as potent bioactive signaling mediators. These metabolites regulate immune cell differentiation and function, modulate angiogenesis and influence inflammatory responses, thereby directly coupling metabolic alterations to tumor–immune interactions^1,5^. Within this framework, CAFs and CAAs have emerged not as inert structural elements but as metabolically active and immunomodulatory hubs. CAFs, which are often the most abundant stromal cell population, commonly adopt a glycolytic phenotype and generate substantial amounts of lactate. Lactate serves as an alternative fuel for tumor cells and suppresses cytotoxic immune activity and promotes angiogenesis^6,7^. Beyond glycolysis, CAFs reprogram glucose, amino acids and lipid metabolism, while secreting paracrine mediators that stimulate tumor proliferation, drive ECM remodeling and facilitate immune escape^6^. CAAs, historically recognized for their role in lipid storage and metabolism, are increasingly recognized as key immunometabolic regulators. Upon receiving tumor-derived signals, CAAs undergo dedifferentiation-like changes, lose their lipid storage capacity, and secrete a repertoire of cytokines, adipokines and fatty acids. These factors rewire immune cell metabolism and foster a tumor-permissive niche that supports cancer growth and metastatic spread^8,9^. Immune cells are deeply embedded in this metabolic network and balance antitumor activity against susceptibility to metabolic suppression. In nutrient-restricted TMEs enriched with immunosuppressive metabolites such as lactate and adenosine, cytotoxic CD8^+^ T lymphocytes (CTLs) and natural killer (NK) cells exhibit reduced proliferation, cytokine production and cytolytic capacity. By contrast, tumor-promoting immune subsets, including M2-polarized macrophages and regulatory T cells (T_reg_), are metabolically advantageous, thereby reinforcing immunosuppressive feedback loops^10^. Ultimately, the reciprocal exchange of metabolites and the resulting metabolic crosstalk among CAFs, CAAs, immune cells and malignant cells sustains the core hallmarks of cancer, including immune evasion, epithelial–mesenchymal transition (EMT), angiogenesis and therapy resistance^6–10^ (Fig. 1).

This Review Article summarizes current insights into the immune metabolic functions of CAFs and CAAs and highlights their influence on immune cell activation, differentiation and effector responses. We further explored the translational potential of disrupting the CAF–CAA–immune cell metabolic crosstalk as a strategy to restore immune competence and enhance the efficacy of existing cancer therapies.

## Metabolic characteristics and CAA-mediated crosstalk

### Definition, origins and functions of CAAs

**Fig. 2:**
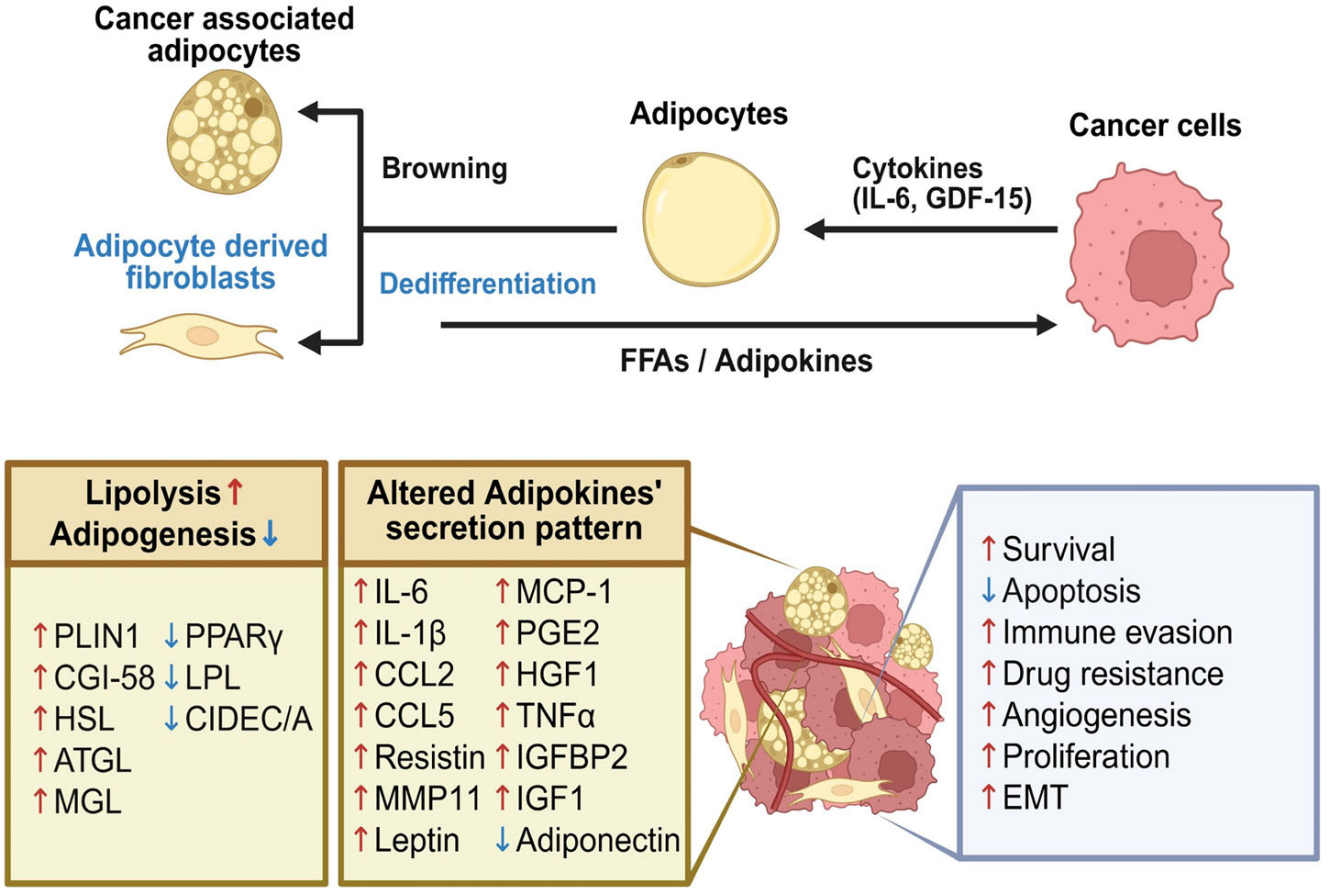
CAA-driven metabolic and paracrine crosstalk with cancer cells. CAAs undergo phenotypic reprogramming marked by suppressed adipogenesis—via downregulation of PPARγ, CEBPA, SREBP1C and CIDEC/CIDEA—and enhanced lipolysis through upregulation of HSL, ATGL and MGL. These changes release FFAs into the TME, fueling oxidative metabolism and biosynthesis in cancer cells. In parallel, CAAs secrete elevated levels of pro-inflammatory, tumor-promoting mediators (for example, IL-6, IL-1β, CCL2, CCL5, MCP-1, PGE₂, HGF and TNF-α) while reducing adiponectin, thereby promoting proliferation, angiogenesis, EMT, immune evasion and therapeutic resistance. Reciprocal signaling from cancer cells induces adipocyte dedifferentiation (adipocyte-derived fibroblasts) and beige-like transdifferentiation (browning), reinforcing a protumorigenic CAA phenotype. Collectively, these processes establish CAAs as dynamic metabolic and signaling hubs that actively sustain tumor progression.

The adipose tissue is a dynamic endocrine organ with far-reaching roles in systemic metabolism, immune regulation and tissue homeostasis. In cancer biology, adipose tissue—particularly peritumoral fat depots—has drawn increasing attention for its active contribution to tumor initiation, progression and metastasis^11,12^. The interaction between adipocytes and malignant cells involves a complex reciprocal exchange of biochemical and biomechanical cues that reprogram adipocyte and tumor cell phenotypes^12,13^. Within this bidirectional signaling network, CAAs emerge as distinct stromal cell populations with specialized metabolic and secretory profiles that actively remodel the TME^14,15^. CAAs most commonly originate from mature white adipocytes that undergo reprogramming following direct contact with tumor cells or exposure to tumor-derived paracrine factors^16,17^. This phenotypic shift is driven by elevated levels of pro-inflammatory and hypoxia-associated mediators, including tumor necrosis factor-α (TNF-α), interleukin-1β (IL-1β), interleukin-6 (IL-6), prostaglandin E₂ (PGE₂) and hypoxia-inducible factor 1α (HIF-1α). Morphologically, CAAs exhibit reduced and dispersed lipid droplets, increased mitochondrial density and expanded rough endoplasmic reticulum features, which are reminiscent of beige adipocytes in both form and function. At the molecular level, they display downregulation of adipogenic and lipid storage-related genes (for example, peroxisome proliferator-activated receptor gamma (PPARγ), fatty acid-binding protein 4 (FABP4), lipoprotein lipase (LPL) and adiponectin) alongside upregulation of pro-inflammatory cytokines, ECM-remodeling enzymes and pro-angiogenic factors. Transcriptional reprogramming is frequently reinforced by epigenetic modifications, including DNA methylation and histone alterations, which may stabilize the CAA phenotype and sustain its tumor-promoting functions over time. Although transdifferentiation from mature white adipocytes is the predominant pathway for CAA formation, lineage-tracing and fate-mapping studies have revealed additional origins. These include differentiation of mesenchymal stem cells, stromal vascular fraction-derived preadipocytes and bone marrow-derived progenitors that infiltrate metastatic niches^18,19^ (Fig. 2).

## Metabolic characteristics of CAAs

### Enhanced lipolysis and fatty acid secretion

CAAs undergo extensive metabolic reprogramming, most prominently through persistent lipolysis activation. This process is driven by the upregulation of adipose triglyceride lipase (ATGL), hormone-sensitive lipase (HSL) and monoacylglycerol lipase (MGL), which sequentially hydrolyze triglycerides into FFAs and glycerol. Lipolytic flux is further amplified by increased expression of co-activators, such as comparative gene identification-58 (CGI-58) and perilipin-1 (PLIN1), coupled with the downregulation of G0/G1 switch gene 2 (G0S2), an endogenous inhibitor of ATGL activity. The FFAs released from CAAs are enriched in monounsaturated and polyunsaturated fatty acids, particularly oleic and linoleic acids, which are readily oxidized by tumor cells. This metabolic coupling is reinforced by cancer cell upregulation of key fatty acid β-oxidation enzymes, including carnitine palmitoyltransferase 1A (CPT1A), carnitine palmitoyltransferase 2 (CPT2) and long-chain acyl-CoA dehydrogenase (ACADL)^16,20–22^. Stable isotope tracing and Seahorse extracellular flux analyses have shown that tumor cells cocultured with CAAs exhibit markedly increased mitochondrial respiration and strong reliance on lipid-driven oxidative phosphorylation (OXPHOS)^14,20^, underscoring the central role of CAA-derived lipids in sustaining tumor bioenergetics under metabolic stress.

### Altered adipokine and cytokine secretion

CAAs display a profound shift from a homeostatic adipokine profile to a pro-inflammatory tumor-promoting secretome. Leptin production is markedly elevated via HIF-1α stabilization, supporting cancer stem cell maintenance through activation of Notch and Signal transducer and activator of transcription 3 (STAT3) signaling pathways. IL-6, which is also upregulated, activates STAT3 in tumor cells and induces programmed death-ligand 1 (PD-L1) expression, thereby enhancing resistance to immune surveillance. TNF-α engages nuclear factor kappa-light-chain-enhancer of activated B cells (NF-κB) signaling, inducing anti-apoptotic proteins such as B-cell lymphoma 2 and cellular inhibitor of apoptosis protein. By contrast, adiponectin levels are considerably downregulated in CAAs. This loss suppresses AMP-activated protein kinase (AMPK) signaling while hyperactivating the mechanistic target of rapamycin (mTOR), thereby favoring anabolic tumor metabolism^13,16,19^^.^ Furthermore, CAA-derived exosomes enriched with oncogenic miRNAs (for example, miR-21 and miR-155) and bioactive lipids serve as intercellular messengers that reinforce tumor-promoting signaling and metabolic reprogramming^14,15^.

### Oxidative stress and hypoxia induction

The peritumoral adipose niche is frequently hypoxic, driving metabolic reprogramming in CAAs through coordinated activation of HIF-1α and HIF-2α. These transcription factors promote glycolytic flux by inducing hexokinase 2 (HK2) and lactate dehydrogenase A (LDHA), while concurrently suppressing the mitochondrial oxidative capacity. In parallel, CAAs generate elevated levels of reactive oxygen species (ROS) through NADPH oxidase 4 activation and mitochondrial dysfunction. ROS accumulation activates redox-sensitive transcription factors such as nuclear factor erythroid 2-related factor 2 and activator protein 1, triggering antioxidant and stress response programs. Although these adaptations promote tumor cell survival under oxidative stress, they also compromise cytotoxic immune cell function, foster immune tolerance and enable the long-term persistence of malignant clones^12,20^.

## Metabolic crosstalk between CAAs and cancer cells

### Lipid mobilization from CAAs, mechanisms of lipolysis and fatty acid trafficking

A hallmark of CAAs is sustained lipolytic activation induced by tumor-derived cytokines and TME hypoxia. Tumor-secreted IL-6, TNF-α and catecholamines enhance HSL and ATGL expression and activity in adjacent adipocytes, catalyzing triacylglycerol (TAG) hydrolysis into FFAs and glycerol^21,22^. CAAs also upregulate PLIN1, CGI-58 and phosphorylated HSL (Ser660), which increases lipid droplet accessibility and accelerates lipid release^21,22^^,^. Concurrently, tumor-derived factors suppress lipid droplet–coating proteins (for example, cell death-inducing DFFA-like effector A (CIDEA) and cell death-inducing DFFA-like effector C (CIDEC)), destabilizing lipid storage, and sustain FFA mobilization^22^. Liberated FFAs accumulate within the TME and are absorbed by tumor cells via transporter-mediated uptake or vesicular delivery^22^. Notably, CAAs secrete extracellular vesicles (EVs) enriched with FFAs, lipogenic enzymes (for example, fatty acid synthase (FASN) and acetyl-CoA carboxylase1 (ACC)), and regulatory microRNAs that induce fatty acid oxidation (FAO) genes, including CPT1A and fatty acid-binding proteins 4 (FABP4)^23^. By providing both metabolic substrates and transcriptional modulators, EVs enhance tumor metabolic flexibility and resilience under nutrient stress, thereby directly contributing to cancer progression and therapy resistance.

### Fatty acid uptake mechanisms, CD36- and FABP4-mediated transport in tumor cells

Tumor cells use multiple pathways to acquire exogenous FFAs, with CD36 (fatty acid translocase), fatty acid transport proteins (FATP1-6) and fatty acid-binding proteins (FABPs) as principal mediators. CD36 is overexpressed in aggressive malignancies, such as breast and pancreatic cancers, and facilitates long-chain fatty acid uptake via clathrin-dependent endocytosis, directing lipids toward the endoplasmic reticulum or mitochondria^23–25^. In addition to its transporter function, CD36 acts as a signaling receptor that activates the Src family kinases and mitogen-activated protein kinase (MAPK) pathways, thereby promoting proliferation, migration, and cancer stem cell maintenance^24,25^. CD36-positive tumor cells exhibit heightened metastatic potential and therapeutic resistance, whereas pharmacological blockade with antibodies or small molecules (for example, sulfosuccinimidyl oleate) markedly reduced tumor growth in preclinical models^23,25^. FABP4, which is enriched in adipocyte-dense microenvironments, functions as an intracellular chaperone that traffics FFA to the mitochondria for oxidation or to lipid droplets for storage. High FABP4 expression is characteristic of tumor metastasis to lipid-rich sites, such as the bone marrow and omentum, in breast and prostate cancers. Silencing FABP4 disrupts lipid-supported mitochondrial respiration and reduces invasive capacity^24^, underscoring its essential role in adipocyte-tumor lipid exchange.

### Integration into mitochondrial β-oxidation and TCA cycle flux

Once internalized, CAAs-derived FFAs are esterified to fatty acyl-CoA by acyl-CoA synthetase long-chain family members (ACSL1/3/4), transported into the mitochondrial matrix via the carnitine shuttle (CPT1A/B, CPT2) and oxidized through β-oxidation. This catabolic process yields acetyl-CoA, NADH and FADH₂, which feed the tricarboxylic acid (TCA) cycle and OXPHOS to sustain ATP production. FAO confers metabolic flexibility, enabling tumor cells to survive glucose deprivation or hypoxia by preserving mitochondrial respiration, maintaining a redox balance and sustaining stem-like phenotypes^26,27^. In triple-negative breast cancer (TNBC), FAO is transcriptionally activated through peroxisome proliferator-activated receptor gamma coactivator 1-α (PGC1α)–PPARα coactivation, which limits ROS accumulation^26,27^. Pharmacological CPT1A inhibition (for example, etomoxir and ST1326) impairs FAO and suppresses tumor growth in preclinical models. Stable isotope tracing with U-¹³C-palmitate reveals that a substantial proportion of TCA cycle intermediates in metastatic breast cancer cells originates from exogenous FFAs^26,28^. Moreover, FAO-derived acetyl-CoA supports NADPH generation via isocitrate dehydrogenase 2 (IDH2)-mediated reverse flux, supplying reducing power for oxidative stress defense^26^.

### Regulation via lipid-activated transcription factors

In addition to serving as structural components and energy substrates, lipids act as potent signaling molecules that regulate transcription via lipid-sensing nuclear receptors. Key regulators include peroxisome proliferator-activated receptors (PPARα, PPARδ and PPARγ), liver X receptor (LXR) and estrogen-related receptor alpha (ERRα), all of which can be activated by fatty acid ligands^28–30^. In breast and ovarian cancers, CAA-derived lipids activate PPARδ, inducing FAO-related genes (for example, CPT1A and acyl-CoA oxidase 1), angiogenic mediators (for example, vascular endothelial growth factor (VEGF)) and inflammatory enzymes (for example, cyclooxygenase-2). PPARγ, typically central to adipogenesis, is aberrantly co-opted in certain cancers to drive lipid droplet biogenesis and metabolic rewiring. ERRα, a master regulator of oxidative metabolism, is stabilized by fatty acid intermediates and deacetylated by sirtuin-1 (SIRT1), enhancing mitochondrial biogenesis and coupling FAO to OXPHOS. Elevated ERRα activity correlates with tumor aggressiveness and resistance to glycolysis-targeted therapies. Pharmacologic targeting of these transcription factors, such as GSK3787 for PPARδ and XCT790 for ERRα, has demonstrated antitumor efficacy in FAO-dependent malignancies^31,32^. These findings highlight their potential as therapeutic nodes in lipid-derived oncogenic programs.

### Tumor type-specific dependency on CAA-derived lipids

Although lipid uptake and oxidation pathways are broadly upregulated in solid tumors, the extent of dependency on CAA-derived lipids varies according to tumor type and microenvironmental context^33,34^. In breast cancer, high CD36 expression and direct adipocyte proximity are strongly correlated with increased metastatic risk and recurrence^32,33^. Ovarian cancer spheroids metastasizing to the omentum overexpress FABP4 and LPL, enabling rapid lipid scavenging from the omental fat^24,28^. Lipid acquisition from CAAs is a critical adaptation in pancreatic ductal adenocarcinoma (PDAC), in which dense desmoplasia restricts vascular nutrient delivery. PDAC cells exhibit high expression of lipophagy-related genes (for example, autophagy-related protein 5, ATGL) and depend on lipid droplet turnover to sustained proliferation^14,21^. By contrast, glioblastoma (GBM) multiforme arises in a lipid-scarce brain environment and relies primarily on de novo lipogenesis rather than exogenous lipid uptake^14,35^.

### Lipid-driven induction of EMT, cancer stemness and metastasis

**Fig. 3:**
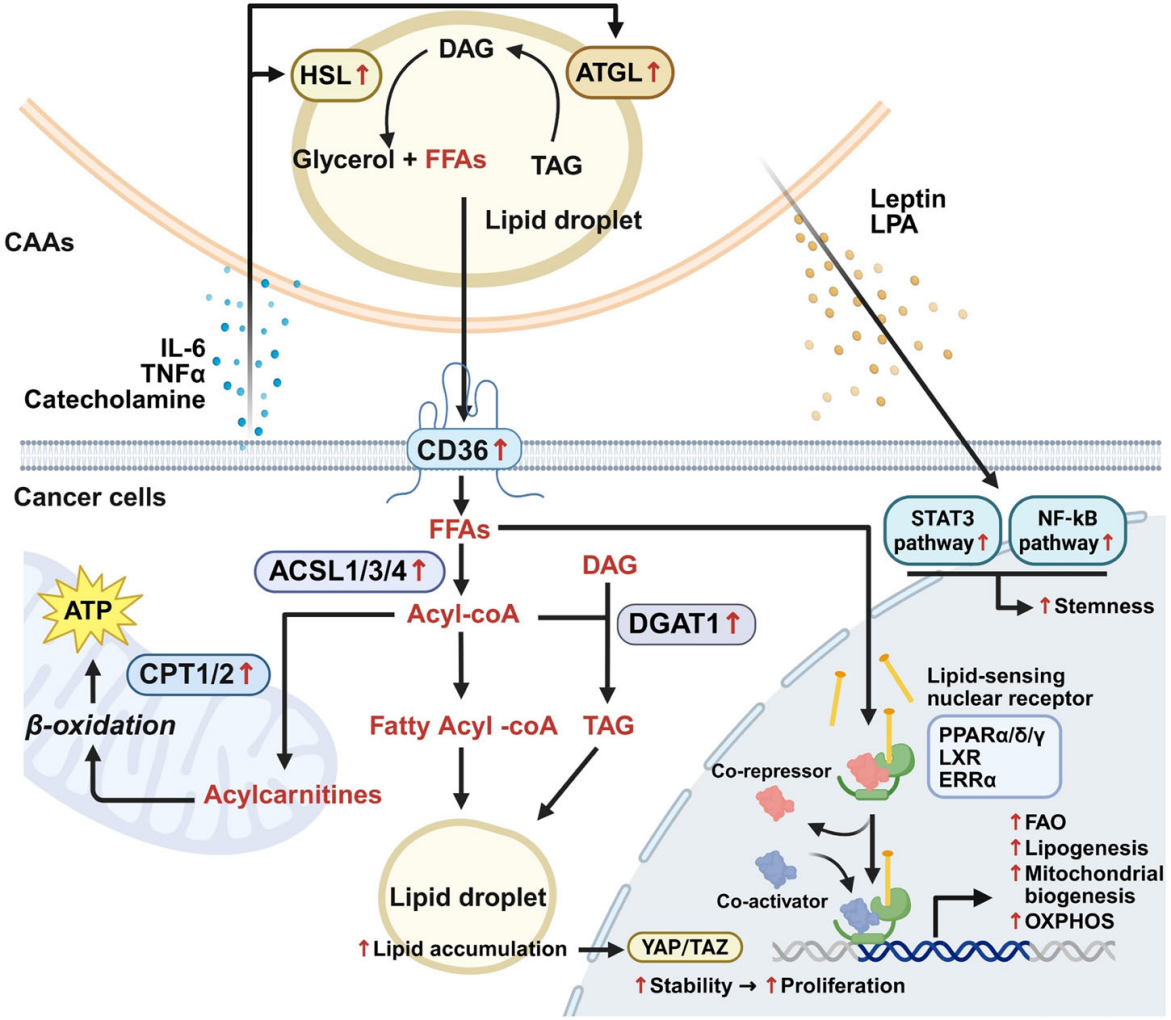
Lipid trafficking from adipocytes to cancer cells and its metabolic signaling consequences. CAAs release glycerol and free FFAs from lipid droplets by activating ATGL and HSL. These FFAs are taken up by cancer cells, mainly through CD36. Inside cancer cells, FFAs are converted into acyl-CoA by ACSL1/3/4, which are either directed to β-oxidation via CPT1/2 to generate ATP or re-esterified into TAG through DGAT1, leading to lipid droplet accumulation. Elevated FFAs also act as ligands for lipid-sensing nuclear receptors (PPARα/PPARδ/PPARγ, LXR and ERRα), which undergo conformational changes, release co-repressors, recruit co-activators and transcriptionally promote FAO, lipogenesis, mitochondrial biogenesis and OXPHOS. Leptin and LPA secreted from CAAs activate the STAT3 and NF-κB pathways in cancer cells, thereby enhancing stemness. In addition, lipid accumulation within cancer cells increases the stability of YAP/TAZ, thereby promoting cancer cell proliferation.

CAA-derived lipids actively promote EMT, stemness acquisition and metastatic dissemination through multiple signaling axes. Palmitate and oleate activate transforming growth factor-beta (TGF-β) and Wnt/β-catenin pathways, inducing EMT transcription factors Snail family transcriptional repressor 1, Twist family BHLH transcription factor 1 and zinc finger E-box-binding homeobox 1^36^. Lipid accumulation also stabilizes YAP/TAZ in the nucleus, a process facilitated by altered membrane mechanics and loss of cell–cell adhesion. Furthermore, CAA-secreted leptin and lysophosphatidic acid (LPA) activate STAT3 and NF-κB pathways, upregulating stemness-associated markers (aldehyde dehydrogenase 1, SRY-box transcription factor 2 and octamer-binding transcription factor 4) and enhancing mammosphere formation. This lipid-driven reprogramming fosters therapeutic resistance, facilitates recurrence and sustains tumor-initiating cell populations, establishing CAA-derived lipids as the central drivers of malignant progression^37–39^ (Fig. 3).

## Metabolic crosstalk between CAAs and immune cells

### CD8^+^ T cells

**Fig. 4:**
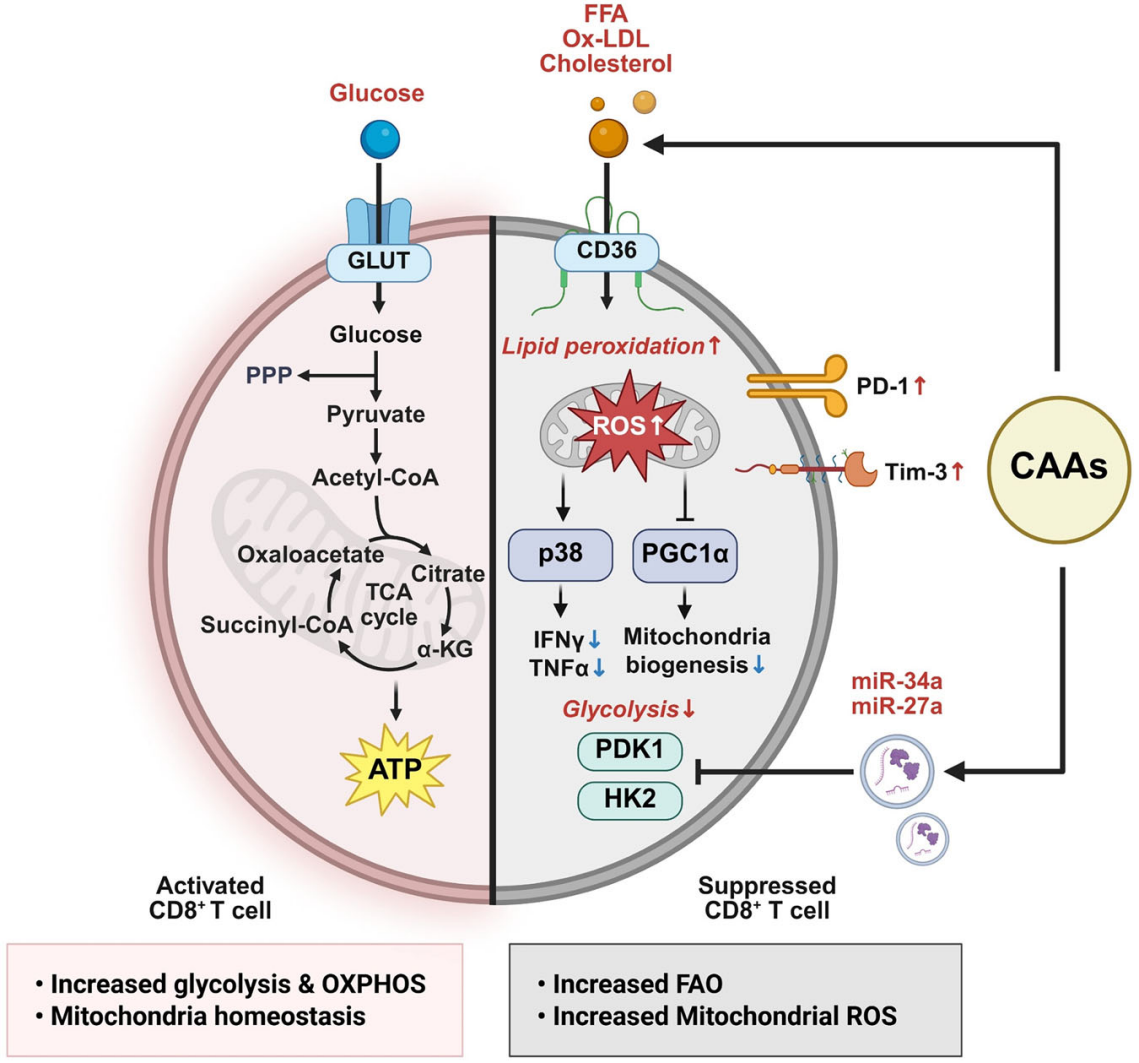
CAA-driven metabolic suppression of CD8^+^ T cells via lipid transfer and mitochondrial stress. Under normal activation, CD8^+^ T cells import glucose via GLUTs to fuel glycolysis, the TCA cycle and mitochondrial homeostasis, supporting robust cytokine production and cytotoxicity. However, in CAA-rich niches, fatty acids, ox-LDL and cholesterol released by CAAs are taken up by fatty acid translocase CD36, redirecting metabolism toward lipid utilization. This shift induces lipid peroxide and reactive ROS accumulation, suppresses mitochondrial biogenesis via PGC1α and activates p38 MAPK, collectively reducing IFN-γ and TNF-α production. Concurrently, the glycolytic capacity is diminished through the downregulation of PDK1 and HK2, whereas CAA-derived microRNA-34a and microRNA-27a further reinforce glycolytic inhibition. This metabolic rewiring culminates in CD8^+^ T cell dysfunction, characterized by reduced effector cytokine output and elevated expression of the exhaustion markers PD-1 and Tim-3, ultimately facilitating tumor immune evasion.

Upon antigen stimulation, effector CD8^+^ T cells undergo a metabolic shift from OXPHOS to aerobic glycolysis, cytokine production and cytolytic activity. The ‘Warburg effect of immunity’ is tightly regulated under physiological conditions but is profoundly disrupted within the TME by nutrient competition, hypoxia and stromal-derived inhibitory cues, particularly CAAs^40^. CAAs actively reshape the metabolic landscape by releasing abundant FFA via heightened lipolytic activity driven by ATGL and HSL. Although tumor cells readily exploit these FFAs to sustain β-oxidation and ATP production, infiltrating CD8^+^ T cells in lipid-enriched niches exhibit profound metabolic impairment. Upregulation of CD36, a long-chain fatty acid transporter, drives excessive FFA, oxidized low-density lipoprotein (ox-LDL) and cholesterol uptake, and skews T cell metabolism toward FAO. Although FAO can sustain basal ATP levels, it fails to meet the anabolic demands of robust effector function, resulting in reduced expression of granzyme B, interferon-gamma (IFN-γ) and perforin, and diminished antitumor activity. Notably, CD36-deficient mouse models restore glycolytic flux and enhance tumor clearance, underscoring the central role of CAA-derived lipids in T cell suppression^41,42^. In addition to inhibiting glycolysis, CAA-derived lipids induce mitochondrial dysfunction and promote T cell exhaustion. Saturated FFAs, particularly palmitate, trigger mitochondrial depolarization and excessive ROS generation, which upregulates inhibitory checkpoint receptors (for example, programmed cell death protein 1 (PD-1), T cell immunoglobulin and mucin-domain containing-3 (TIM-3) and lymphocyte activation gene-3 (LAG-3)) while suppressing PGC1α, a master regulator of mitochondrial biogenesis. This results in a metabolically exhausted CD8^+^ T cell phenotype characterized by impaired respiratory capacity. Spatial single-cell RNA sequencing of adipocyte-rich tumor regions revealed CD8^+^ T cells co-expressing FAO-associated genes and exhaustion markers, indicating that CAAs drive a distinct immunometabolic exhaustion state beyond classical antigen-driven mechanisms^42,43^. In addition, CAAs secrete EVs enriched with bioactive lipids, metabolic enzymes such as FASN and ACC, and regulatory microRNAs (for example, miR-27a and miR-34a). These vesicles impair glycolysis by downregulating key metabolic regulators (HK2 and pyruvate dehydrogenase kinase 1 (PDK1)) and alter lipid raft composition via ceramide and sphingolipid transfer, thereby disrupting T cell receptor signaling^44^ (Fig. 4).

### NK cells

CAAs profoundly modulate NK cell function within the TME through metabolic, cytokine-mediated and immune checkpoint-linked mechanisms. Dedifferentiated CAAs release abundant FFAs and enhance lipid uptake in NK cells via transporters such as SR-B1 and CD36, leading to intracellular lipid droplet accumulation, metabolic shift toward FAO and suppression of cytotoxicity and IFN-γ production^45,46^. CAA-derived adipokines, including IL-6, leptin and PGE₂, further impair NK cell effector functions by downregulating perforin and granzyme B while upregulating exhaustion markers such as PD-1, TIM-3 and LAG-3^19,47^. In obesity-associated CAAs, these effects are amplified, with metabolic stress reinforcing checkpoint expression and epigenetic remodeling of the effector gene loci^48,49^.

### MDSCs

CAAs release a cocktail of bioactive molecules and growth factors, such as G-CSF, which orchestrates myeloid-derived suppressor cells (MDSC) mobilization, metabolic reprogramming and functional enhancement. Leptin and FFAs from CAAs activate the JAK–STAT3 pathway in MDSCs, increasing the production of immunosuppressive mediators, including arginase, nitric oxide and ROS, which inhibit T cell activity and promote tumor immune evasion^50^. CAAs also secrete chemokines, such as CCL2, recruiting CCR2^+^ monocytes into tumors, where they differentiate into immunosuppressive MDSCs and M2 macrophages. These recruited myeloid populations amplify local immunosuppression, reshape the tumor stroma and blunt T cell-mediated antitumor responses. CAA-derived G-CSF plays a dual role: facilitating tumor cell EMT through STAT3 activation and mobilizing granulocytic MDSCs into the TME, thereby linking metastatic programming to immune evasion. Similarly, CAA-secreted CXCL8 (IL-8) contributes to the recruitment of immunosuppressive myeloid cells, reduces T cell infiltration and promotes PD-L1 expression in tumors, all of which reinforce the immunosuppressive niche^50–52^. Beyond recruitment, CAAs induce metabolic rewiring on MDSCs. MDSCs absorb abundant lipids from CAAs, store them as triglyceride droplets and shift their metabolism toward FAO^52,53^. This metabolic adaptation enhances MDSC survival, proliferation and suppressive capacity, thereby consolidating their immunoregulatory phenotypes in the fatty TME. Notably, tumor genetic alterations, such as p53 loss, further prime this axis by reprogramming neighboring adipocytes into immunomodulatory CAAs^54^. These adipocytes secrete inflammatory cytokines, attract Ly6C^+^ and Ly6G^+^ MDSCs, and elevate PD-L1 expression, thereby intensifying immune suppression and resistance to therapy.

### T_reg_

T_reg_, particularly the CD4 ^+^CD25 ^+^FoxP3 ^+^ subset, maintain immune tolerance under homeostatic conditions. However, within the TME, selective enrichment and heightened suppressive capacity play major roles in immune evasion. T_reg_ preferentially accumulate in adipocyte-rich tumor niches, including the omentum in ovarian cancer, PDAC and breast adipose tissue in TNBC^55,56^. This enrichment correlated with elevated levels of FFAs, adipokines and cytokines secreted by CAAs, implicating lipid-mediated signaling as a central driver of T_reg_ expansion, stabilization and suppressive potency. T_reg_ display exceptional metabolic plasticity, favoring oxidative metabolism (predominantly FAO) over glycolysis. This preference enables them to outcompete effector T cells (T_eff_), which depend heavily on glucose and are thus susceptible to nutrient scarcity in the TME^56^. At the molecular level, T_reg_ adaptation to lipid-rich environments is orchestrated by PPARγ, a lipid-sensing nuclear receptor highly expressed in T_reg_. Unsaturated FFAs, such as oleic and linoleic acids, which are released from CAAs, are imported via CD36 and FABP5, activating PPARγ. Activated PPARγ forms a heterodimer with the retinoid X receptor (RXR) and binds to PPAR response elements (PPREs) in the promoters of immunoregulatory genes^56^. This transcriptional program reinforces FoxP3 expression and upregulates suppressive mediators, including cytotoxic T-lymphocyte-associated protein 4, interleukin-10 (IL-10) and T cell immunoreceptors with Ig and ITIM domains. PPARγ-driven FoxP3 stability is further strengthened by STAT5 activation downstream of IL-2 receptor signaling, enabling T_reg_ to retain suppressive function even in pro-inflammatory conditions. In murine models, T_reg_-specific PPARγ deficiency leads to impaired immunosuppression and reduced tumor growth, confirming the indispensable role of lipid sensing in CAA-fueled T_reg_-mediated immune suppression^56^.

### TAMs

Tumor-associated macrophages (TAMs) are among the most abundant immune cell populations in solid tumors and exhibit remarkable functional plasticity within the TME^57^. Macrophages range from tumoricidal pro-inflammatory M1-like cells to immunosuppressive protumor M2-like cells^58^. In adipocyte-rich malignancies such as breast, ovarian and pancreatic cancers, TAMs are predominantly skewed toward an M2-like phenotype, characterized by elevated expression of arginase-1 (Arg1), CD206, IL-10 and TGF-β^59^. CAAs play a pivotal role in driving this polarization through the release of lipid mediators, adipokines and EVs, which converge on key signaling hubs, signal transducer and activator of transcription 6 (STAT6), PPARγ and mTOR, while reprogramming macrophage metabolism toward FAO and OXPHOS^60,61^. Metabolically, M2-like macrophages preferentially rely on FAO and mitochondrial respiration, whereas M1-like macrophages depend on aerobic glycolysis and inducible nitric oxide synthase-driven nitric oxide production^57^. In vitro, CAA-conditioned macrophages display a marked increase in the oxygen consumption rate, CPT1A upregulation and lipid droplet accumulation^58^. These changes are fueled by the uptake of CAA-derived FFAs via CD36 and the scavenger receptor-A^59^. Once internalized, FFAs are converted to acyl-CoA and catabolized through β-oxidation, generating acetyl-CoA not only for ATP synthesis but also as a substrate for histone acetylation at M2-associated gene loci (Arg1, IL-10 and Mannose receptor C-type 1). This epigenetic remodeling reinforces the M2 transcriptional program^62^. Disruption of FAO reprograms TAMs toward an M1-like phenotype and restores IL-12 production either pharmacologically with an etomoxir or genetically via CPT1A silencing, underscoring the central role of lipid metabolism in CAA-driven immunosuppressive polarization^57^. At the transcriptional level, this process is co-governed by PPARγ and STAT6. PPARγ, activated by CAA-derived monounsaturated fatty acids and prostaglandin derivatives, binds to enhancers of M2-associated genes (Arg1, Mrc1 and Chitinase-like protein 3) in concert with histone acetylation^59,63^. STAT6 is activated by elevated IL-4 and interleukin-13 (IL-13) in adipocyte-rich TMEs^57^ and signals through Interleukin-4 receptor subunit alpha and IL-13 receptor subunit alpha-1 to induce Janus kinase 1/3 phosphorylation, STAT6 dimerization and nuclear translocation^58^. In the nucleus, STAT6 co-occupies M2 gene loci alongside PPARγ, synergistically amplifying transcriptional output, a cooperation validated by chromatin immunoprecipitation sequencing in adipocyte-conditioned macrophages^60,63^.

## Metabolic characteristics and CAF-mediated crosstalk

### Definition, origins and functions of CAFs

CAFs are among the most abundant and persistently activated stromal cell populations in the TME, exerting profound effects on tumor progression, metastasis and immune modulation^61^. Unlike quiescent normal fibroblasts, CAFs remain chronically activated and are commonly identified by markers such as α-smooth muscle actin (α-SMA), fibroblast activation protein (FAP) and platelet-derived growth factor receptors alpha/beta, although no universal marker exists^64^. CAFs have diverse cellular origins, including tissue-resident fibroblasts, mesenchymal stem cells, epithelial or endothelial cells undergoing mesenchymal transition, adipocyte precursors and pericytes. This heterogeneity in ontogeny underpins the substantial functional diversity across tumor types^65^. Functionally, CAFs remodel the TME through the secretion of growth factors (for example, TGF-β and HGF), cytokines (for example, IL-6 and IL-8) and ECM components, thereby promoting cancer cell proliferation, invasion, angiogenesis and metabolic adaptation^66^. A defining hallmark of CAF biology is its ability to engage in metabolic crosstalk with tumor cells and supply metabolites such as lactate, pyruvate and lipids to support the bioenergetic and biosynthetic demands^67^. Beyond metabolic support, CAFs actively contribute to immune evasion by restricting T cell infiltration, skewing macrophage polarization toward an immunosuppressive M2-like phenotype and enhancing T_reg_ function. In some contexts, CAFs can present antigens via major histocompatibility complex class II molecules, thereby directly influencing immune cell recruitment and activation^68,69^.

## Metabolic characteristics of CAFs

### Glycolytic reprogramming and lactate production

CAFs frequently undergo aerobic glycolysis even under normoxic conditions, mirroring the Warburg effect observed in cancer cells. This glycolytic shift is driven primarily by HIF-1α stabilization, TGF-β signaling and tumor-derived exosomal cues^70^. The upregulation of glycolytic enzymes, including HK2, pyruvate kinase M2 (PKM2) and phosphofructo-2-kinase/fructose-2,6-bisphosphatase 3 (PFKFB3), facilitates glucose uptake and conversion to lactate.

Lactate is exported via the monocarboxylate transporter 4 (MCT4) into the TME, where it is absorbed by cancer cells through monocarboxylate transporter 1 (MCT1) to fuel mitochondrial OXPHOS. This ‘reverse Warburg effect’ not only spares glucose for anabolic biosynthesis in cancer cell but also promotes EMT, angiogenesis and immune suppression^67^.

## Amino acid metabolism and nutrient exchange

### Glutamine–glutamate dynamics

A hallmark of CAF amino acid metabolism is its role in regulating glutamine and glutamate fluxes in the TME. Under nutrient-scarce conditions, CAFs upregulate glutamine synthetase to synthesize glutamine de novo from glutamate and ammonium^71^, an essential adaptation when cancer cells have limited access to extracellular glutamine. In ovarian cancer, CAF-derived glutamine fuels tumor cell proliferation by supplying carbon and nitrogen for nucleotide biosynthesis and maintaining redox homeostasis. A reciprocal metabolic loop emerges in which tumor cells release glutamate, which is taken up by CAFs and recycled into glutamine. This glutamine–glutamate cycle exemplifies cooperative nutrient sharing that sustains tumor growth. In PDAC, Netrin G1-positive CAFs display high glutamine synthetase and vesicular glutamate transporter 1 expression, further amplifying glutamine availability. In addition to metabolic provisioning, CAFs enhance the secretion of immunosuppressive cytokines, thereby coupling nutrient support with immune regulation^72^. By safeguarding amino acid availability under metabolic stress, CAFs buffer tumor cells against nutrient deprivation while reinforcing immune suppression.

### Proline biosynthesis for ECM production

Proline, a non-essential amino acid, is indispensable for collagen synthesis and a defining function of CAFs. Type I collagen, which is enriched in proline and hydroxyproline, requires a continuous supply of proline to sustain ECM deposition. To meet this requirement, CAFs upregulate pyrroline-5-carboxylate reductase 1 (PYCR1), which catalyzes the conversion of glutamate-derived α-ketoglutarate to proline via the ornithine pathway^73^. In breast cancer models, PYCR1-driven proline biosynthesis is essential for maintaining a collagen-rich ECM. Genetic or pharmacological inhibition of PYCR1 in CAFs markedly reduced collagen content and importantly impaired tumor invasion and metastasis, highlighting the metabolic–structural axis by which amino acid metabolism drives cancer progression. Moreover, mitochondrial pyruvate dehydrogenase-mediated generation of acetyl-CoA supports histone acetylation at collagen gene promoters, reinforcing the epigenetic programming of CAFs toward a profibrotic, ECM-producing phenotype.

### Other amino acids

In glutamine-depleted TMEs, CAFs activate alternative nitrogen-donating pathways to sustain both their biosynthetic needs and those of the adjacent tumor cells. A key adaptation is the upregulation of asparagine synthetase, driven by activating transcription factor 4 (ATF4), during the integrated stress response^74^. In prostate cancer, metabolically stressed CAFs secrete asparagine, which is subsequently absorbed by tumor cells to support protein synthesis and activate mTOR signaling. Asparagine production is frequently coupled with pyruvate carboxylase activity, underscoring the existence of a nitrogen-sharing network between CAFs and cancer cells that buffers tumors against nutrient scarcity. In addition to asparagine, other amino acids are integral to the CAF–tumor metabolic axis. Cystine uptake via the membrane transporter SLC7A11, followed by intracellular reduction to cysteine, sustains glutathione pools in CAFs, enabling both CAFs and tumor cells to withstand oxidative stress^75^. Moreover, certain CAF subsets express Arg1, which depletes local arginine, thereby inhibiting T cell proliferation and effector function and directly contributing to immunosuppression within the TME^76^. Collectively, these amino acid-mediated interactions demonstrate that CAF metabolism is not merely a nutrient supply line for tumor growth but also a critical orchestrator of ECM remodeling and immune modulation.

### Lipid metabolism and lipid droplet dynamics

CAFs undergo profound lipid metabolic reprogramming, which actively sustains tumor progression. Unlike quiescent fibroblasts, CAFs markedly upregulate key lipogenic enzymes, most notably FASN and stearoyl-CoA desaturase-1 (SCD1). Newly synthesized fatty acids are sequestered within lipid droplets and subsequently mobilized for transfer to the adjacent cancer cells. In recipient tumor cells, these fatty acids act as energy-dense substrates that fuel mitochondrial β-oxidation, sustain survival under metabolic stress and enhance metastatic potential^77^. In addition to serving as metabolic fuel, CAF-derived lipids also function as potent paracrine oncogenic mediators. LPA, a bioactive lipid secreted by CAFs, activates PI3K/AKT signaling in cancer cells, thereby promoting proliferation, migration and therapy resistance^78^. In pancreatic and breast cancers, elevated LPA production is accompanied by lipid droplet depletion in activated CAFs, reflecting a metabolic switch from lipid storage toward lipid mobilization. CAF-driven lipid metabolism also facilitates malignant phenotypes by promoting EMT and cancer stemness, partly through modulation of PPAR signaling and plasma membrane remodeling. Specific lipid species influence the ECM architecture by regulating collagen crosslinking enzymes and fibroblast contractility, thereby coupling lipid metabolism to the mechanical reinforcement of the tumor stroma.

## Metabolic crosstalk between CAFs and cancer cells

### Glycolytic symbiosis and lactate shuttle

CAFs consistently adopt an aerobic glycolytic phenotype characterized by elevated glucose uptake and conversion to lactate. This reprogramming is driven by tumor-derived cues, including TGF-β and oxidative stress, and hypoxia and is marked by upregulation of glycolytic enzymes such as HK2, PFKFB3 and PKM2^79^. Lactate generated by CAFs is exported via MCT4 and imported into cancer cells via MCT1. Once internalized, lactate is oxidized in the TCA cycle to fuel mitochondrial OXPHOS, thereby reducing the dependence of cancer cells on glucose^80^. This lactate shuttle establishes a metabolic symbiosis in which CAFs act as lactate suppliers and tumor cells as efficient lactate consumers. By preserving glucose for anabolic biosynthesis, cancer cells sustain biomass production, proliferation and survival under nutrient-limited conditions. Concurrently, the lactate-driven acidification of the TME suppresses cytotoxic immune activity, promotes ECM degradation and facilitates cell invasion.

### Pyruvate exchange and redox homeostasis

In addition to lactate, glycolytic CAFs secrete substantial amounts of pyruvate into the TME through MCT4. Under metabolic or oxidative stress, cancer cells import pyruvate through MCT1 to sustain mitochondrial respiration and maintain the redox balance^81^. Once internalized, pyruvate serves two critical roles: (1) as a TCA cycle substrate under glucose or glutamine restriction, pyruvate fuels oxidative metabolism and sustains ATP production. (2) Electron acceptor for NAD^+^ regeneration—via lactate dehydrogenase (LDH)—pyruvate accepts electrons from NADH, regenerating NAD^+^ to preserve glycolytic flux. This NAD^+^ recycling is particularly vital in hypoxic or drug-compromised tumors where electron transport chain activity is impaired^82^. In PDAC and lymphoma models, CAF-derived pyruvate mitigates ROS accumulation and confers resistance to mitochondrial inhibitors such as phenformin. Moreover, pyruvate can stabilize HIF-1α, reinforcing glycolysis in both CAFs and cancer cells. This reciprocal exchange creates a redox-buffered nutrient-sharing niche that supports long-term tumor persistence and therapeutic resistance.

### Lipid transfer and lipogenic signaling

**Fig. 5:**
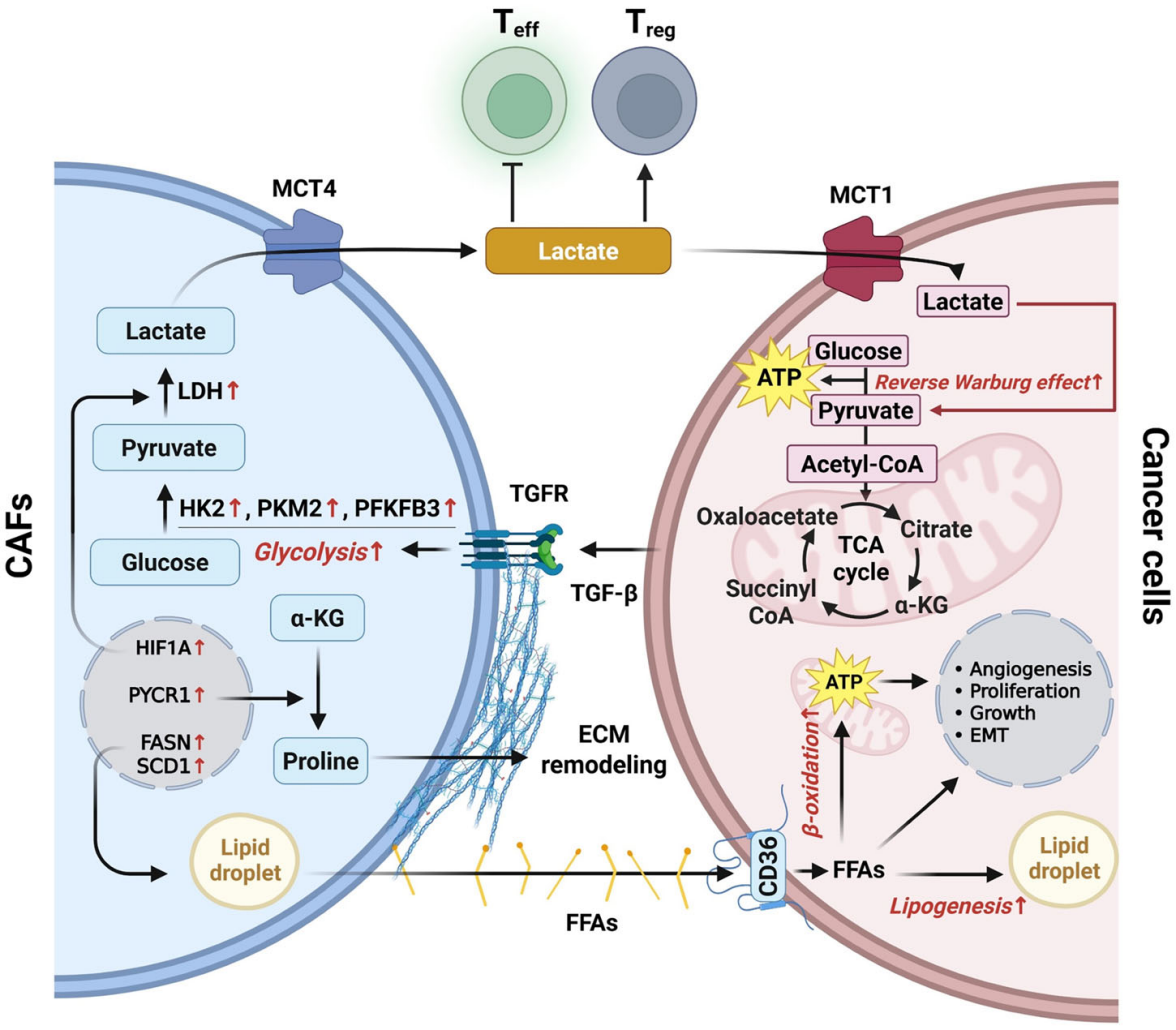
Cancer cell-derived TGF-β drives glycolytic reprogramming in CAFs and establishes a reverse Warburg effect-mediated metabolic symbiosis. TGF-β released by cancer cells activates surrounding fibroblasts into CAFs and induces the expression of key glycolytic enzymes—HK2, PKM2 and PFKFB3—thereby enhancing glucose catabolism and lactate production via LDH. Lactate is exported through MCT4 and subsequently imported into cancer cells via MCT1, which fuels the TCA cycle and OXPHOS. This ‘reverse Warburg effect’ enables glucose sparing in cancer cells for anabolic pathways such as lipid and glutamine biosynthesis. In parallel, CAFs facilitate ECM remodeling through proline biosynthesis, driven by PYCR1 upregulation and α-ketoglutarate metabolism, while transferring fatty acids via CD36 to support cancer cell β-oxidation. These coordinated metabolic exchanges sustain tumor proliferation, EMT, angiogenesis and therapeutic resistance. Moreover, lactate accumulation in the TME suppresses T_eff_ function, while stabilizing T_reg_ and reinforcing immune evasion.

CAFs undergo profound lipid metabolic reprogramming, functioning as both a reservoir of metabolic substrates and a source of lipid-derived signaling molecules. Upregulation of lipogenic enzymes such as FASN and SCD1 with newly generated fatty acids stored in lipid droplets before mobilization^80^. Cancer cells acquire CAF-derived FFAs through transporter such as CD36 or FABP4, subsequently oxidizing them via mitochondrial β-oxidation. Lipid influx is particularly advantageous under glucose-deprived or hypoxic conditions as it sustains OXPHOS, preserves energy homeostasis and promotes cancer stem-like traits. In addition to their bioenergetic roles, CAF-derived lipids act as potent paracrine effectors. In breast and colorectal cancers (CRCs), LPA secreted by CAFs activates PI3K/AKT signaling, driving proliferation, migration and therapeutic resistance. In PDAC, lipids released from the activated pancreatic stellate cells, which are quiescent precursors of CAFs, play dual roles as metabolic substrates and oncogenic signals. CAF-derived lipids can remodel cancer cell plasma membranes, facilitating invadopodia formation and ECM degradation, thereby enhancing invasiveness. Pharmacological inhibition of FASN or SCD1 in CAFs disrupts this lipid-fueled metabolic signaling axis, attenuating tumor growth and metastasis. Collectively, these findings suggest that CAF–tumor metabolic crosstalk is a highly integrated system, wherein glycolytic and lipid-based exchanges not only satisfy cancer cell bioenergetic demands but also drive oncogenic signaling, immune evasion and therapy resistance, making these pathways compelling therapeutic targets within the TME (Fig. 5).

## Metabolic crosstalk between CAFs and immune cells

### CD8^+^ T cells

The highly glycolytic phenotype of CAFs drives excessive glucose consumption, depleting extracellular glucose and limiting its availability in T cells. This nutrient competition suppresses glycolytic flux in CTLs, reducing production of key effector molecules, including IFN-γ, granzyme B and TNF-α^83^. Under hypoxic TME conditions, CAFs further exacerbate metabolic suppression through HIF-1α-mediated upregulation of glucose transporter 1 (GLUT1) and glycolytic enzymes such as PFKFB3 and LDHA. The resulting lactate is exported via the MCT4 transporters, which further exacerbates glucose deprivation. Accumulated extracellular lactate inhibits T cell receptor signaling, prevents the nuclear translocation of NFAT, attenuates mTOR activity and induces intracellular acidification, collectively impairing T cell proliferation and effector cytokine release^84^. In parallel, CAF-derived TGF-β synergistically with lactate to drive T cell exhaustion, characterized by elevated expression of immune checkpoint receptors PD-1, TIM-3 and LAG-3, alongside downregulation of IL-7 receptor (CD127), thereby compromising T cell survival and memory formation^85^. The metabolic constraints imposed by CAFs also influence CD4^+^ T cell lineage specification. The T_H_1 and T_H_17 subsets are glycolysis-dependent, whereas T_H_2 cells and T_reg_ preferentially utilize OXPHOS and FAO. By creating a glucose-depleted niche, CAFs selectively suppress T_H_1/T_H_17 polarization, while favoring T_reg_ expansion. Moreover, CAF-derived IL-6, prostaglandin E2 (PGE_2_) and VEGF induce CD4^+^ T cell differentiation toward T_H_2 or functionally inert states. In addition to glucose metabolism, CAFs modulate amino acid availability to reinforce immunosuppression. Through the expression of indoleamine 2,3-dioxygenase (IDO1), CAFs catabolize tryptophan to kynurenine, an immunosuppressive metabolite that activates the aryl hydrocarbon receptor (AhR) in CD4^+^ T cells, promoting T_reg_ differentiation at the expense of effector lineages. Furthermore, in response to IFN-γ from activated T cells, CAFs upregulate PD-L1, which engages PD-1 on CD4^+^ T cells to inhibit PI3K/AKT signaling, suppress glycolysis and induce cell cycle arrest. CAF-derived galectin-1 further consolidates this immunosuppressive program by binding to glycosylated receptors on CD4^+^ T cells and triggering apoptosis or functional inactivation^86^.

### NK cells

CAFs are the central architects of the TME and actively impair NK cell antitumor function through a combination of soluble mediators, ECM remodeling and metabolic reprogramming. Recent studies demonstrate that CAFs secrete immunosuppressive cytokines, such as TGF-β and IL-6, which blunt NK cell cytotoxicity by downregulating activating receptors including NKG2D and NKp30, while simultaneously inducing expression of inhibitory checkpoint molecules (PD-1, TIM-3) on NK cells. In parallel, CAF-derived PGE2 and IDO metabolites alter NK cell metabolism, limiting their production of IFN-γ and perforin^87,88^. CAFs also indirectly influence NK cell activity through ECM remodeling. Dense collagen deposition and cross-linking reduce NK cell infiltration into tumor nests, creating a physical barrier that hinders immune surveillance^89^. Emerging data have highlighted the metabolic crosstalk as another suppressive axis. CAFs release lactate and lipids, shifting NK cell metabolism toward exhaustion-like states characterized by diminished glycolytic capacity^87–91^. This metabolic stress, coupled with the CAF-induced upregulation of inhibitory ligands such as PD-L1, reinforces immune evasion.

### MDSCs

CAFs strongly influence expansion, recruitment and immunosuppressive activity of MDSCs. CAFs secrete a broad spectrum of soluble mediators, including CXCL12, CCL2 and IL-6, which drive the chemotaxis of immature myeloid cells into tumors and promote their differentiation into MDSCs. CXCL12–CXCR4 signaling not only enhances MDSC accumulation but also spatially organizes them in stromal niches, where they effectively block T cell infiltration^92,93^. In addition to chemokines, CAF-derived IL-6 and GM-CSF activate STAT3 signaling in MDSCs, a central pathway that sustains their survival and enhances their suppressive functions. This signaling cascade augments the production of arginase-1, nitric oxide and ROS, which collectively inhibit T cell proliferation and cytotoxicity. CAF-secreted TGF-β further reinforces this immunosuppressive loop by skewing MDSCs toward a more regulatory phenotype and suppressing NK and CD8^+^ T cell activity^94–96^. Metabolic crosstalk plays a crucial role in this process. CAFs release lactate and other metabolites that reprogram MDSCs toward glycolysis and FAO, increasing their persistence and suppressive potency within the nutrient-depleted TME.

### T_reg_

CAFs play an indispensable role in shaping a T_reg_-permissive niche through direct substrate provision, nutrient landscape remodeling and redox regulation. Activated CAFs upregulate FASN and lipolytic enzymes such as ATGL and HSL to liberate FFAs, including palmitate and oleate, into the TME. Notably, oleic acid augments FoxP3 expression and mitochondrial respiration, reinforcing the suppressive phenotype. The CAF expression of lipid-binding proteins, including FABP4 and FABP5, further facilitates efficient lipid trafficking to proximate T_reg_, sustaining their metabolic demands even under hypoxic or glucose-restricted conditions. In parallel, CAFs export large quantities of lactate through MCT4 as a byproduct of elevated glycolytic flux. Although T_eff_ are metabolically incapacitated in lactate-rich environments, T_reg_ maintain their function via MCT1-mediated lactate uptake. Once internalized, lactate fuels the TCA cycle, supports NAD^+^ regeneration and sustains oxidative metabolism, allowing ATP production despite glucose scarcity^97^. Lactate also promotes T_reg_ chemotaxis via CCR8 upregulation and stabilizes FoxP3 expression through HIF-1α-dependent transcription, facilitating their accumulation and functional dominance in hypoxic tum or regions. CAFs also modulate T_reg_ redox homeostasis via cysteine metabolism. Through SLC7A11-mediated cystine import and subsequent cysteine release, CAF help to buffer oxidative stress. In addition, CAF-derived TGF-β and IL-33 enhance mitochondrial biogenesis and ROS scavenging in T_reg_, reinforcing their OXPHOS-dependent metabolic program. In addition to lipid and redox regulation, CAFs directly promote T_reg_ lineage commitment via amino acid metabolism. In response to IFN-γ, CAFs upregulate IDO, catalyzing tryptophan degradation to kynurenine. Kynurenine activates AhR in T cells, driving T_reg_ differentiation and stabilizing FoxP3 expression.

### TAMs

CAFs function as central metabolic and signaling hubs that dictate TAM polarization and activity through nutrient competition, metabolite provision, cytokine secretion and ECM remodeling^98^. However, in CAF-rich tumor regions, elevated glycolytic activity in CAFs depletes extracellular glucose, thereby depriving M1 macrophages of a critical energy source. Simultaneously, CAF-derived lactate accumulates in the TME and is imported by macrophages via monocarboxylate transporters, where it stabilizes HIF-1α, suppresses inducible nitric oxide synthase expression and reduces IL-12 production, collectively impairing M1 polarization and effector capacity^68,99^. By contrast, CAFs actively foster M2-like polarization, a phenotype dependent on FAO and glutaminolysis. FFAs released by CAFs through lipolysis and transported via FABP4 and CD36 are readily taken up by macrophages and oxidized to fuel OXPHOS. In parallel, CAF-derived glutamine is converted to α-ketoglutarate, activating mTORC1 and inducing epigenetic remodeling at M2-associated gene loci, including *Arg1, Mrc1* (CD206) and *Vegf*. CAF-secreted cytokines such as IL-6, IL-10 and TGF-β further engage STAT3 and SMAD signaling cascades, consolidating the M2 transcriptional program^100^. In addition to soluble mediators, CAFs exert biomechanical control over the TAM phenotype through ECM remodeling. By depositing collagen, enriching hyaluronan and inducing lysyl oxidase (LOX)-mediated crosslinking, CAFs generate a dense, stiff stroma that engages integrin receptors and activates YAP/TAZ signaling in macrophages, further skewing them toward an M2-like state. Moreover, CAF-derived chemokines, including CXCL12 and CCL2, recruit monocytes and retain TAMs within tumor-promoting niches. Importantly, CAF-educated TAMs have been implicated in resistance to immune checkpoint blockade, positioning them as critical therapeutic barriers and high-value targets for stroma–immune comodulation strategies^101,102^.

### Therapeutic strategies targeting CAFs and CAAs in the TME

**Fig. 6:**
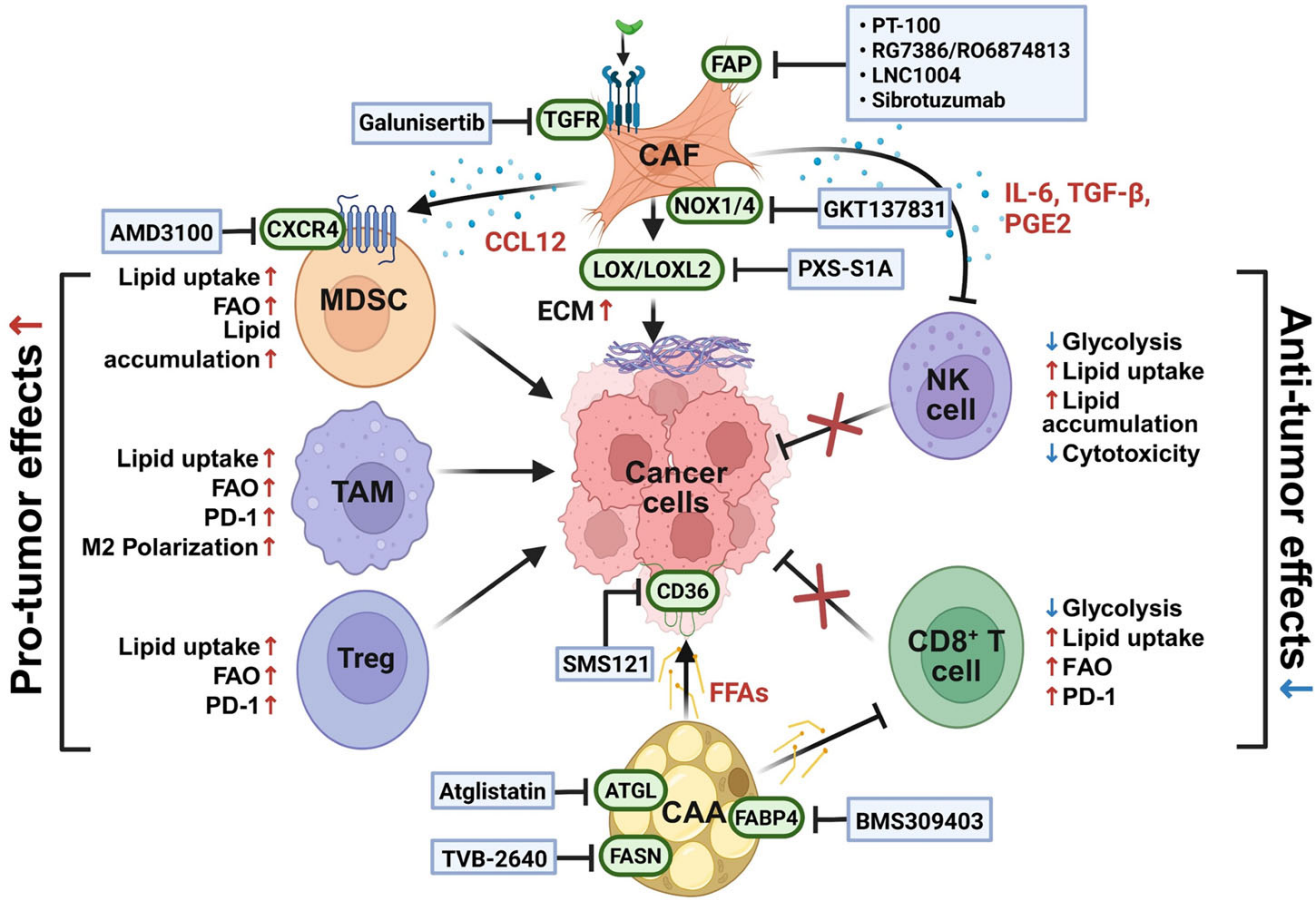
Metabolic crosstalk among CAAs, CAFs and immune cells in the TME and therapeutic targets. A schematic illustration of complex metabolic and immunomodulatory interactions within the TME. CAAs FFA via ATGL and FASN, which are transported through FABP4 and taken up by cancer cells (via CD36), MDSCs, TAMs and T_reg_, fueling FAO and promoting immunosuppressive phenotypes (Increased M2 polarization, PD-1 expression and lipid accumulation). CAFs remodel the ECM through LOX/LOXL2 and secrete IL-6, TGF-β and PGE2, further enhancing tumor progression and impairing NK and CD8^+^ T cell cytotoxicity by reducing glycolysis and boosting lipid uptake/FAO. Key inhibitors targeting this network are indicated: ATGL (atglistatin), FASN (TVB-2640), FABP4 (BMS309403), CD36 (SMS121), NOX1/4 (GKT137831), LOX/LOXL2 (PXS-S1A), TGFR (galunisertib) and FAP (PT-100, RG7386/RO6874813, LNC1004 and sibrotuzumab). Together, these interventions aim to normalize the TME, reduce immunosuppression and restore antitumor immunity.

Targeting stromal components is emerging as a compelling strategy for reprogramming the immunosuppressive TME and enhancing its anticancer efficacy. Both CAFs and CAAs function as central metabolic and immunological hubs, fueling tumor progression through nutrient provisioning, secretion of bioactive metabolites and suppression of antitumor immunity. Consequently, therapeutic interventions that disrupt these stromal-driven processes are gaining momentum in preclinical models and early phase clinical trials. Therapeutic approaches against CAFs can be broadly categorized into three main strategies: (1) direct depletion of tumor-promoting CAF subsets to reduce their protumorigenic influence, (2) phenotypic reprogramming of CAFs toward quiescent or tumor-restraining states, thereby restoring tissue homeostasis, (3) inhibition of CAF-mediated ECM remodeling and immunosuppressive signaling, targeting pathways such as TGF-β, CXCL12/CXCR4 and focal adhesion kinase (FAK) (Table 1). Similarly, CAAs represent a metabolically reprogrammed stromal population that promotes tumor progression through enhanced lipolysis, the release of fatty acids and adipokines and the induction of oxidative stress. Potential strategies to target CAAs included: (1) blocking lipid mobilization and uptake pathways (for example, CD36, FABP4 and CPT1A) to prevent fatty acid fueling of tumor cells, (2) inhibiting pro-inflammatory cytokines and adipokines with tumor-promoting activity (for example, IL-6 and leptin), (3) modulating lipid-sensing nuclear receptors such as PPARγ and ERRα to counteract adipocyte-driven metabolic reprogramming in both cancer cells and immune infiltrates (Table 2). A persistent challenge lies in the absence of robust biomarkers capable of distinguishing between tumor-promoting and tumor-restraining stromal subtypes. This limitation raises concerns regarding off-target effects of indiscriminate stromal depletion. Moving forward, selective targeting guided by functional and metabolic phenotyping, spatial transcriptomics and single-cell profiling is essential to define precise therapeutic windows and minimize collateral tissue damage. Thus, targeting the metabolic and immunomodulatory functions of CAFs and CAAs holds substantial translational promise. Realizing this potential requires a deeper mechanistic understanding of stromal–immune–cancer cell crosstalk and the rational design of personalized, combination-based regimens capable of dismantling stromal-mediated support systems that sustain tumor growth and therapy resistance (Fig. 6).

**Table 1:** Overview of clinical trials targeting CAFs for cancer treatment.

Drugs/chemicals	Classification	Combination therapy	Therapeutic effects	Cancer models	Preclinical/clinical	Reference
PT-100 (Val-boro-Pro)	FAP inhibitor	None	Inhibition of FAP activity	Metastatic CRC	Phase II	^103,104^
RG7386/ RO6874813	FAP and Death receptor5 bispecific antibody	None	Expensed effector T and NK cells without T_reg_	Renal cell carcinoma	Phase 1	^105^
ESC11 and ESC14	Human Fab immunoconjugates	Radiotherapy	Reduced tumor growth	Melanoma	Preclinical	^106^
LNC1004	Albumin-binding FAP radiopharmaceutical	None	High tumor-localized radiation, promising efficacy	Metastatic RAIR thyroid cancer	Phase II	^107^
OMTX705	FAP targeted Antibody drug conjugate (ADC)	Gemcitabine/paclitaxel	Enhanced CD8^+^ T cell infiltration and delayed recurrence	Fibrosarcoma	Preclinical	^108^
Sibrotuzumab	anti-FAP monoclonal antibody	None	Improved disease stability in CRC	Metastatic CRC	Phase I/II	^109^
Pirfenidone	Anti-fibrotic non-steroidal anti-inflammatory drug	Cisplatin	Reduced tumor–stroma crosstalk and tumor progression	Non-small cell lung cancer	Preclinical	^110^
Imatinib mesylate	Platelet-Derived Growth Factor Receptor(PDGFR) inhibitor	Buparlisib	Reduced CAF-driven tumor proliferation via PDGF signaling blockade	Lung cancer	Preclinical	^111^
Galunisertib	TGF-β1 receptor inhibitor	Anti-PD-L1 therapy / oxozeaenol	Eliminated metastases, prolonged recurrence-free survival	PDAC	Phase I/II	^112^
Vismodegib	Sonic-hedgehog pathway inhibitor	Abraxane and Doxil	Enhanced drug efficacy	Pancreatic cancer	Phase I/II	^113^
Sonidegib (LDE225)	A selective Smo modulator, a key component of the hedgehog pathway	Docetaxel	Safe in combination, Increased antitumor activity	TNBC	Phase Ib	^114^
ABBV-085	Monomethyl auristatin E-conjugated ADC targeting leucine rich repeat containing protein 15	None	Inhibited tumor growth in vitro and in vivo	Osteosarcoma	Preclinical	^115^
Anakinra	IL-1R antagonist	5-FU/bevacizumab	Reduced CAF-derived thymic stromal lymphopoietin	Metastatic CRC	Phase II	^116^
GKT137831 (Setanaxib)	NOX1/4 inhibitor	None	Restored immunotherapy response in CAF-rich tumors	Lung cancer	Phase II	^117^
AMD3100 (plerixafor)	CXCR4 inhibitor	Anti-PD-1 immunotherapy	Reversed CAF-mediated immunosuppression, enhanced anti-PD-1 response	Multiple myeloma	Phase II	^118^
PXS-S1A	LOX/LOXL2 inhibitor	None	Inhibited tumor growth and angiogenesis	Breast cancer	Preclinical	^119^

**Table 2:** Overview of clinical trials targeting CAAs for cancer treatment.

Drugs/chemicals	Classification	Combination therapy	Therapeutic effects	Cancer models	Preclinical/clinical	Reference
BMS309403	FABP4 inhibitor	Carboplatin	Reduced tumor burden and enhanced carboplatin sensitivity	Ovarian cancer	Preclinical	^120^
TVB-3664	FASN inhibitor	Sulfosuccinimidyl oleate	Synergistic inhibited proliferation of CRC	CRC	Preclinical	^121^
Atglistatin	ATGL inhibitor	None	Suppressed proliferation in various cancer lines	CRC	Preclinical	^122^
Orlistat	FASN inhibitor	Paclitaxel	Induced apoptosis, increased ferroptosis, inhibited angiogenesis	Hepatocellular carcinoma	Preclinical	^123^
SMS121	CD36 inhibitor	None	Impaired lipid uptake, reduced myeloid leukemia cell viability	Acute myeloid leukemia	Preclinical	^124^
pCCL2 trap	CCL2-trapping plasmid in LPD nanoparticles	PD-L1 blockade	Suppressed CAAs, enhanced T cell infiltration, reduced MDSCs and M2 macrophages	TNBC	Preclinical	^52^
TVB-2640 (denifanstat)	FASN inhibitor	Enzalutamide (Enza)/bevacizumab/taxane	Reduced tumor growth, induced apoptosis, cell cycle arrest	Castration-resistant prostate cancer	Phase I/II	^125^
Cerulenin	FASN inhibitor	Sorafenib	Inhibited cancer stem cell-like traits and metastatic potential	Hepatocellular carcinoma stem cells	Preclinical	^125^
Anti-macrophage inflammatory protein 2 (MIP2) antibody	MIP2 specific antibody	Anti-PD-1 antibody	Enhanced antitumor immunity, inhibited tumor progression	TNBC	Preclinical	^53^
pshRNA- angiopoietin-like 4 (ANGPTL4)	Short-hairpin (RNA) targeting ANGPTL4 secreted by adipocytes	Carboplatin	Inhibited metastasis and energy metabolism	Ovarian cancer	Preclinical	^126^
Allo-aca	Leptin receptor antagonist peptide	Cisplatin	Prolonged survival after subcutaneous administration	TNBC	Preclinical	^127^
ACT001	Plasminogen activator inhibitor 1 (PAI-1) inhibitor	Cisplatin	Inhibited proliferation and invasion of GBM via PAI-1/PI3K/AKT pathway	GBM	Phase I	^128^
PF-04136309	CCR2 inhibitor	Nanoparticle albumin-bound paclitaxel and gemcitabine	Reduced TAM infiltration, enhanced sensitivity to therapy	PDAC	Phase I	^129^
Marimastat	Broad-spectrum MMP inhibitor	None	No clear benefit as second-line treatment	Metastatic breast cancer	Phase III	^130^
Etomoxir	CPT1 inhibitor	Temozolomide	Reduced GBM stemness and invasiveness, improved survival	GBM	Preclinical	^131^
AdipoRon	Adiponectin receptor agonist	2-Deoxy glucose/oxamate	Inhibited non-small cell lung cancer growth via cell cycle suppression	Non-small cell lung cancer	Preclinical	^132^

## Conclusion

The TME is sustained by a dynamic web of metabolic exchanges among CAFs, CAAs, immune cells and malignant cells that collectively shape tumor evolution, immune evasion and resistance. CAFs undergo distinct metabolic adaptations, including elevated glycolysis, reprogrammed amino acid utilization and specialized lipid handling, which drive tumor growth, ECM remodeling and suppression of antitumor immunity. Similarly, CAAs display extensive metabolic rewiring, marked by enhanced lipolysis and the release of bioactive lipids and inflammatory mediators, which collectively promote cancer cell proliferation, metastatic dissemination and immune modulation. Within this metabolically altered TME, immune effector populations, such as CTLs and NK cells, are functionally compromised by nutrient depletion and the accumulation of immunosuppressive metabolites, including lactate and adenosine. By contrast, T_reg_ and M2-like TAMs are metabolically adapted to, and even thrive within, the lipid-rich CAF/CAA-conditioned niche, thereby reinforcing immunosuppressive circuits. From a therapeutic standpoint, dismantling stromal–metabolic crosstalk offers a potent means of restoring antitumor immunity and overcoming resistance. The pharmacological inhibition of lipid metabolism can reduce tumor fatty acid uptake, impair cancer cell viability and reverse T cell dysfunction. Blocking lactate transport through MCT1/MCT4 inhibition can attenuate lactate-driven immunosuppression and reinvigorate CTL activity. In addition, modulating transcriptional regulators including PPARγ, STAT3 and HIF-1α holds promise for reversing stromal-driven metabolic reprogramming and re-establishing effective immune surveillance.

Integrating these metabolism-targeting strategies with existing modalities, including immune checkpoint blockade, chemotherapy and radiotherapy, offers a rational path toward synergistic therapeutic efficacy. Such combinatorial, metabolism-informed interventions represent a promising frontier for precision oncology, with the potential to not only overcome drug resistance but also achieve durable disease control and improve patient survival.
